# Modifying Cassava Starch via Extrusion with Phosphate, Erythorbate and Nitrite: Phosphorylation, Hydrolysis and Plasticization

**DOI:** 10.3390/polym16192787

**Published:** 2024-10-01

**Authors:** Phanwipa Wongphan, Cristina Nerin, Nathdanai Harnkarnsujarit

**Affiliations:** 1 Department of Packaging and Materials Technology, Faculty of Agro-Industry, Kasetsart University, 50 Ngam Wong Wan Rd., Latyao, Chatuchak, Bangkok 10900, Thailand; phanwipa.w@ku.th; 2 GUIA Group, Department of Analytical Chemistry, University of Zaragoza, I3A, María de Luna, 3, 50018 Zaragoza, Spain; cnerin@unizar.es; 3 Center for Advanced Studies for Agriculture and Food, Kasetsart University, 50 Ngam Wong Wan Rd., Latyao, Chatuchak, Bangkok 10900, Thailand

**Keywords:** modified starch, cassava starch, food preservative, hydrogen bonding, extrusion

## Abstract

Extrusion processing of plasticized cassava starch, a prominent industrial crop, with chemical additives offers a thermo-mechanical approach to modify starch structures through physical and chemical interactions. This research investigates the interaction and morphology of thermoplastic cassava starch (TPS) blended with tetrasodium pyrophosphate (Na_4_P_2_O_7_), sodium tripolyphosphate (Na_5_P_3_O_10_), sodium hexametaphosphate (Na_6_(PO_3_)_6_), sodium erythorbate (C_6_H_7_O_6_Na), and sodium nitrite (NaNO_2_) via twin-screw extrusion. The effects of these additives on the chemical structure, thermal profile, water absorption, and solubility of the TPS were examined. The high temperature and shearing forces within the extruder disrupted hydrogen bonding at α-(1-4) and α-(1-6) glycosidic linkages within anhydroglucose units. Na_4_P_2_O_7_, Na_5_P_3_O_10_ and Na_6_(PO_3_)_6_ induced starch phosphorylation, while ^1^H NMR and ATR-FTIR analyses revealed that C_6_H_7_O_6_Na and NaNO_2_ caused starch hydrolysis. These additives hindered starch recrystallization, resulting in higher amorphous fractions that subsequently influenced the thermal properties and stability of the extruded TPS. Furthermore, the type and content of the added modifier influenced the water absorption and solubility of the TPS due to varying levels of interaction. These modified starch materials exhibited enhanced antimicrobial properties against *Escherichia coli* and *Staphylococcus aureus* in polyester blends fabricated via extrusion, with nitrite demonstrating the most potent antimicrobial efficacy. These findings suggest that starch modification via either phosphorylation or acid hydrolysis impacts the thermal properties, morphology, and hydrophilicity of extruded cassava TPS.

## 1. Introduction

Cassava (Manihot esculenta Crantz), a vital industrial crop with extensive applications, plays a significant role in food production and industrial products. It finds wide cultivation in tropical and subtropical regions across Asia, Africa, and Latin America, with major producers including Nigeria, Indonesia, Brazil, and Thailand [1,2]. In recent years, cassava starch extrusion has emerged as a promising technology for enhancing the functionality and value of cassava starch. This process involves applying heat, pressure, and mechanical shear to transform the starch into a variety of expanded, modified, and functionalized products. Extrusion offers numerous benefits, including improved digestibility, enhanced solubility, increased viscosity, and expanded applications in the food and non-food industries [2,3,4]. During extrusion, cassava starch is mixed with plasticizers, additives, and other substances. The combined effect of these ingredients and the shear forces within the extruder leads to the transformation, realignment, and reorganization of starch molecules. This restructuring modifies properties like viscosity, solubility, and digestibility [1,4,5]. Additionally, the high temperature and pressure conditions may partially degrade starch molecules, resulting in the formation of anhydroglucose units [2].

Extruded cassava starch offers remarkable versatility, transforming into various forms based on its intended use for various industries. In the food industry, it can take shapes suitable for snacks, soups, and baked goods. Alternatively, it can be processed into pellets, flakes, or specific shapes to function as texturizing agents or functional ingredients. Beyond food applications, extruded cassava starch finds valuable use in non-food sectors like pharmaceuticals, adhesives, and even biodegradable plastic [6,7]. Extruded cassava starch’s versatility makes it an attractive alternative to other starches and hydrocolloids. It offers manufacturers a sustainable and cost-effective solution for product development, unlocking new opportunities across various industries. Cassava starch extrusion is a pivotal technology that enhances functionality and versatility, promoting its utilization in food (snacks, soups, and baked goods), pharmaceuticals, adhesives, and even biodegradable plastics [2,8,9,10]. Cassava starch extrusion presents a powerful technology for innovation and value creation. It allows manufacturers to meet the evolving demands of consumers and industries globally. This study delves into the physical and chemical processes involved. The present study investigates how twin-screw extrusion transforms starch and how the addition of alternative food preservatives, namely tetrasodium pyrophosphate (Na_4_P_2_O_7_), sodium tripolyphosphate (Na_5_P_3_O_10_), sodium hexametaphosphate (Na_6_(PO_3_)_6_), sodium erythorbate (C_6_H_7_O_6_Na) and sodium nitrite (NaNO_2_), affects the starch. These additives are commonly used in the food industry. The incorporation of starch with preservatives is a promising avenue for sustainable food and non-food product processing. This study aims to provide valuable information on creating alternative functional starch products such as food packaging applications. It explores how the type and number of reactive groups in these food preservatives influence the chemical, thermal, and physical properties of extruded starch through physicochemical modification.

## 2. Materials and Methods

### 2.1. Starch Extrusion

Five food preservatives were selected for this study, namely Na_4_P_2_O_7_, Na_5_P_3_O_10_, Na_6_(PO_3_)_6_, C_6_H_7_O_6_Na, and NaNO_2_. All were sourced from a Thai company: Thai Food and Chemical Co., Ltd. (Bangkok, Thailand) (except sodium nitrite, which came from Vicchi Enterprise Co., Ltd., Bangkok, Thailand). These preservatives were incorporated at two concentration levels (2% and 10%, *w*/*w*) into a mixture containing 35% glycerol (*w*/*w*) (a plasticizer obtained from Patum Vegetable Oil Co., Ltd., Bangkok, Thailand). The mixing process utilized a hot-plate stirrer at 90 °C for 90–120 min. The selection of 2 and 10% concentrations of food preservatives was made to study the difference in concentration and to obtain the highest efficiency. In addition, the developed material has the same cost and has increased functions, and still has good processability and formability. In addition, when adding more than 10% sodium nitrite and sodium erythrobate, the extrusion process will swell and become very sticky (high moisture absorption).

Native cassava starch (Siam Modified Starch Co., Ltd., Bangkok, Thailand) was first dried in a hot air oven at 50 ± 2 °C overnight. The dried starch powder was then mixed with the previously prepared food preservative-glycerol mixture using a dough mixer (SC-236A, Stelang, Foshan, China) for 10 min. This starch-preservative-glycerol mixture was subsequently transformed into thermoplastic starch (TPS) using a co-rotating twin-screw extruder (Type LTE20-48, Labtech Engineering, Samut Prakan, Thailand). The extruder had an L/D ratio of 48 and a screw diameter of 20 mm. The relevant extrusion parameters were a torque of 70–80% and die pressure of 160–170 bar. The barrel temperature profile was set to gradually increase from hopper to die (across 12 zones of 95/100/115/125/135/140/145/150/155/155/160/160 °C), with a screw speed of 120 rpm. The resulting extruded TPS was cooled in air, cut into 2.5 mm pellets, and stored in Ziploc bags for further analysis. Before testing, it was kept in a controlled cabinet at 50% relative humidity and 25 °C for 2 days. The extruded TPS with different food preservative additions after extrusion was denoted as native starch, starch-2% and starch-10% of each food preservative, respectively.

### 2.2. Characterization of TPS Extrudates

#### 2.2.1. Nuclear Magnetic Resonance (NMR)

Proton nuclear magnetic resonance (^1^H-NMR) spectroscopy was employed to characterize the samples. Spectra were recorded on an Ascend™ 600/Advance III HD spectrometer (Bruker, Fällanden, Switzerland) operating at a ^1^H resonance frequency of 600 MHz. Chemical shifts were reported in parts per million (ppm). Samples (approximately 14 ± 1 mg) were dissolved in deuterated dimethyl sulfoxide (DMSO-d6) for analysis.

#### 2.2.2. Fourier Transform Infrared Spectrometer (FTIR)

FTIR spectroscopy was employed to analyze the samples. The spectra were recorded on a Bruker Tensor 27 FT-IR Spectrometer (Bruker OPTIK GmbH, Ettlingen, Germany) using the attenuated total reflection (ATR) mode. The wavenumber range scanned was 500–4000 cm^−1^ with a resolution of 4 cm^−1^. Air was used for background subtraction. Each spectrum was the average of 64 co-added scans to improve the signal-to-noise ratio. Triplicate spectra were averaged.

#### 2.2.3. X-ray Diffractometer (XRD)

X-ray diffraction patterns were obtained using a Bruker AXS D8 diffractometer (Bruker AXS, Karlsruhe, Germany). The samples were scanned in the 2 θ range of 4° to 40° with a step size of 0.02° and a scan rate of 0.8° per second. The operating voltage and current were set at 40 kV and 40 mA, respectively. The degree of crystallinity was calculated using MDI Jade 6 software (Materials Data, Inc., Livermore, CA, USA) by determining the ratio between the crystalline and amorphous fractions in the XRD patterns.

### 2.3. Thermal Properties

#### 2.3.1. Differential Scanning Calorimetry (DSC)

Thermal properties were determined using a differential scanning calorimeter (DSC 1, STAR^e^ system, Mettler Toledo, Greifensee, Switzerland). Samples 5.5–9.5 mg were sealed in an aluminum pan. The heating profiles were recorded in three steps. The first step was heated from −80 to 200 °C with a heating rate of 10 °C/min, and the second step was a determined cooling process using temperatures from 200 to −80 °C and a cooling rate of 10 °C/min. The final step was heated from −80 to 250 °C with a heating rate of 10° C/min. All samples were analyzed under a nitrogen atmosphere with a flow rate of 25 mL/min.

#### 2.3.2. Thermogravimetric Analysis (TGA)

Samples were determined for degradation temperature using thermogravimetric analysis (TGA 2 STAR^e^ System, Mettler Toledo, Greifensee, Switzerland). Samples weighing approximately 10–20 mg were placed in an aluminum pan and then positioned within a ceramic crucible cup. The samples were heated under a nitrogen atmosphere (flow rate of 20 mL/min) from 25 °C to 900 °C at a heating rate of 10 °C/min.

### 2.4. Water Absorption Capacity

Water absorption was determined based on a modified method from Nisitthichai, et al. [11] and Zhang, et al. [12]. Briefly, 1 g samples of the extrudates were immersed in 10 mL of distilled water at room temperature (25 ± 2 °C) for various time intervals: 30, 60, 120, 180, 240, and 300 min. The samples were then carefully removed and excess surface water was blotted using filter paper before being weighed again. The water absorption percentage was calculated from triplicate samples using Equation (1).

(1) $$\mathrm{Water}\;\mathrm{absorption}\;\mathrm{capacity}\;(\%)=\frac{weight\;of\;sample\;at\;time\;(g)-weight\;of\;sample\;at\;initial\;time\;\left(t=0\right)(g)}{weight\;of\;sample\;at\;initial\;time\;\left(t=0\right)(g)}\times 100\%$$

### 2.5. Water Solubility

Water solubility was determined following the method described by Zhang, Shao, Wan, Zhang, Cai, Hu, and Duan [12]. Briefly, 1 g samples of the extrudates were dried in a hot air oven at 70 °C for 24 h. After drying, the samples were stored in a desiccator containing silica gel for 1 h and weighed to obtain the initial dry mass. Subsequently, the samples were immersed in 10 mL of distilled water and placed in a water bath maintained at room temperature (25 ± 2 °C) for varying time intervals: 30, 60, 120, 180, 240, and 300 min. After each immersion time, the samples were recovered, dried again in a hot air oven at 70 °C for 24 h, cooled in a desiccator for 1 h, and weighed. The water solubility percentage was calculated based on the mass loss from triplicate samples using Equation (2).

(2) $$\mathrm{Water}\;\mathrm{solubility}\;(\%)=\frac{initial\;dried\;sample\;weight\;\left(g\right)-final\;dried\;sample\;weight\;\left(g\right)}{initial\;dried\;sample\;weight\;\left(g\right)}\times 100\%$$

### 2.6. Applications of TPS Extrudates

The TPS extrudates were transformed into active films by blending them with poly(butylene adipate-co-terephthalate) (PBAT) via single-screw blown film extrusion following the protocol described by Wongphan, et al. [13]. The antimicrobial activity of the active films was determined by measuring the total plate count of *Escherichia coli* ATCC 25922 and *Staphylococcus aureus* ATCC 25923 after 24 h of incubation at 37 °C. This was done using a modified version of the method described by Laorenza and Harnkarnsujarit [14], which included the microbial strains *Escherichia coli* and *Staphylococcus aureus* obtained from the Department of Microbiology, Faculty of Science, Kasetsart University, Thailand. We cut 1 g of film samples into strips and dissolved them in 1 mL of NB medium (HiMedia Laboratories Pvt. Ltd., Thane, India) containing the bacterial strains *Escherichia coli* and *Staphylococcus aureus*. The NB medium was then diluted with 9 mL of 0.1% peptone solution. We spread 1 mL aliquots of the mixed solutions (at dilutions of 10^−4^ and 10^−5^) on nutrient agar (NA, HiMedia Laboratories Pvt. Ltd., Thane, India) and incubated them at 37 °C for 24 h. The results were expressed as log CFU/mL.

### 2.7. Statistical Analysis

Statistical analysis was performed using IBM SPSS Statistics 22 software (IBM Corporation, Somers, NY, USA). One-way analysis of variance (ANOVA) was followed by Duncan’s multiple range test to identify significant differences among the means of treatment groups. A significance level of α = 0.05 (*p* < 0.05) was used.

## 3. Results and Discussion

### 3.1. X-ray Diffractometer (XRD)

**Figure 1:**
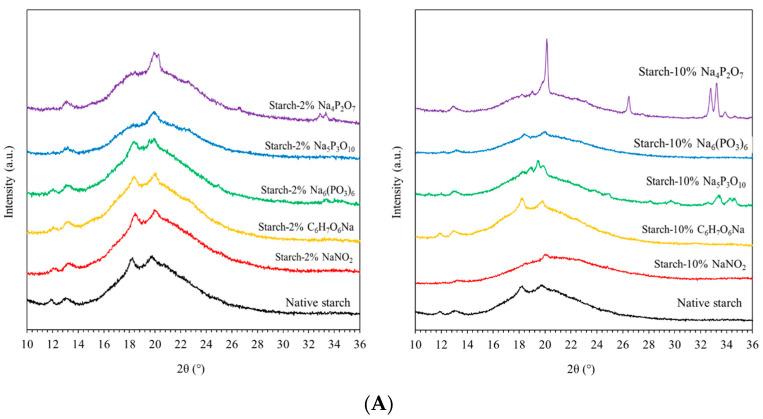

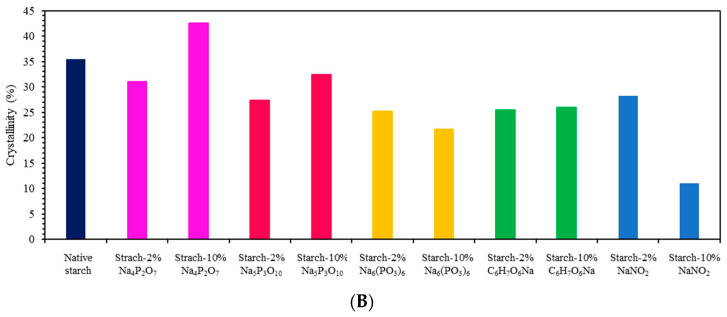
(**A**) The XRD diffractograms and (**B**) the degree of crystallinity of native starch and starch extrudates containing 2 and 10% of food preservatives, namely tetrasodium pyrophosphate (Na_4_P_2_O_7_), sodium tripolyphosphate (Na_5_P_3_O_10_), sodium hexametaphosphate (Na_6_(PO_3_)_6_), sodium erythorbate (C_6_H_7_O_6_Na), and sodium nitrite (NaNO_2_).

Figure 1A depicts the XRD patterns of native starch and starch extrudates containing various food preservatives. The native cassava starch exhibited an A- or C_A_-type polymorphism with characteristic peaks at 2 θ ≈ 15.1°, 17.1°, 18.1°, 26.3°, 30.3°, and 33.0° [15,16]. These polymorphs represent compact monoclinic envelopes and hexagonal unit cells [15,16]. Thermal extrusion is known to induce complex starch gelatinization and amylose recrystallization during cooling. These processes involve chain conformation, crystal packing, and chain alignment [3,15]. The XRD patterns indicate evidence of this recrystallization with a strong peak at 2 θ ≈ 19.8° and a smaller peak at 2 θ ≈ 13.0°, corresponding to the V-type polymorph [17]. This V-polymorph originates from single-helical glucopyranosyl chains induced by thermal processing and starch retrogradation [3,15,17].

The native starch extrudate displayed diffraction peaks at around 2 θ ≈ 12.0°, 13.0°, 18.2°, and 19.8°, indicating a crystalline arrangement containing A-, C_A_-, and V-type polymorphs. The presence of the V-polymorph suggests thermal extrusion processing and possible starch retrogradation [3,15,17]. The incorporation of 10% Na_4_P_2_O_7_ and Na_5_P_3_O_10_ resulted in new diffraction peaks at 2 θ ≈ 26.5°, 32.7°, 33.2°, and 34.6°. Additionally, these samples exhibited the highest peak intensity at 2 θ ≈ 19.8°, likely due to the combined effects of high heating during extrusion, plasticization by glycerol, and the specific food preservatives, which may influence chain ordering and recrystallization. In contrast, starch extrudates containing 10% of Na_6_(PO_3_)_6_, C_6_H_7_O_6_Na, and NaNO_2_ displayed a decrease in peak intensity, suggesting a reduction in crystallinity (Figure 1B). This decrease in crystallinity ranged from 12% to 28% upon incorporation of 2–10% food preservatives. The order of crystallinity for the starch extrudates was as follows: Na_4_P_2_O_7_ (42.57%) > Na_5_P_3_O_10_ (32.45%) > C_6_H_7_O_6_Na (26.00%) > Na_6_(PO_3_)_6_ (21.65%) > NaNO_2_ (11.07%). The observed decrease in crystallinity with certain food preservatives (Na_5_P_3_O_10_ and Na_6_(PO_3_)_6_) might be attributed to interactions with starch such as crosslinking or acid hydrolysis, respectively [18,19]. These interactions could potentially lead to longer starch conformations and hinder chain alignment, ultimately modifying amylopectin and amylose chain structures [5,7,20].

### 3.2. Fourier Transform Infrared Spectrometer (FTIR)

**Figure 2:**
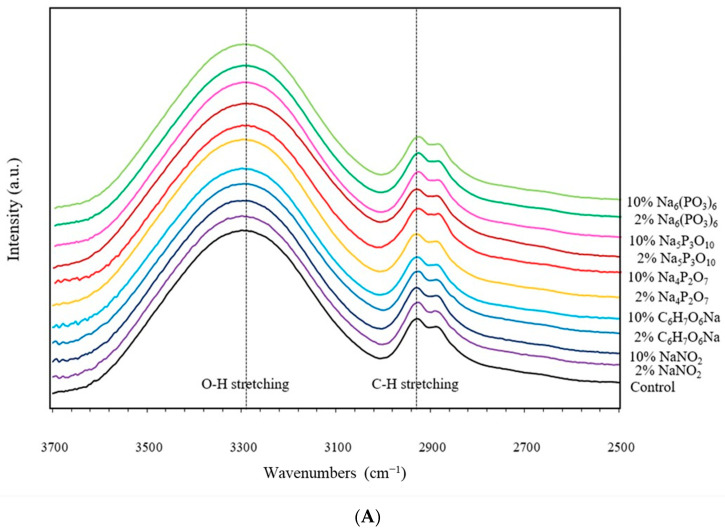

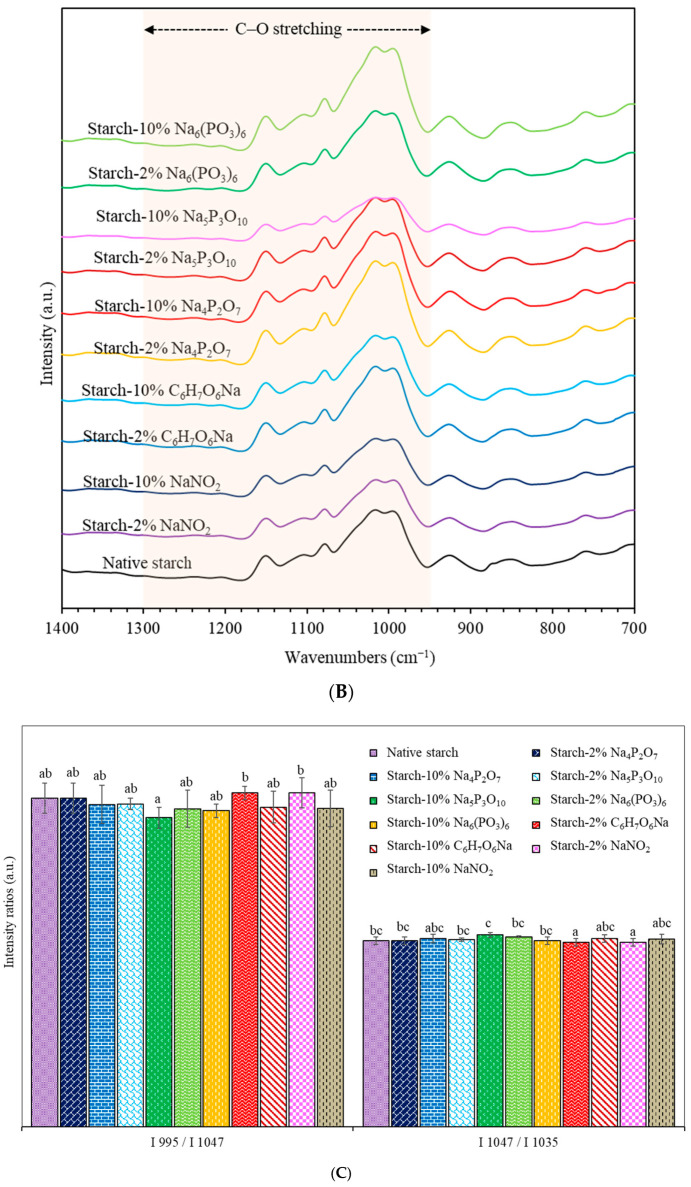

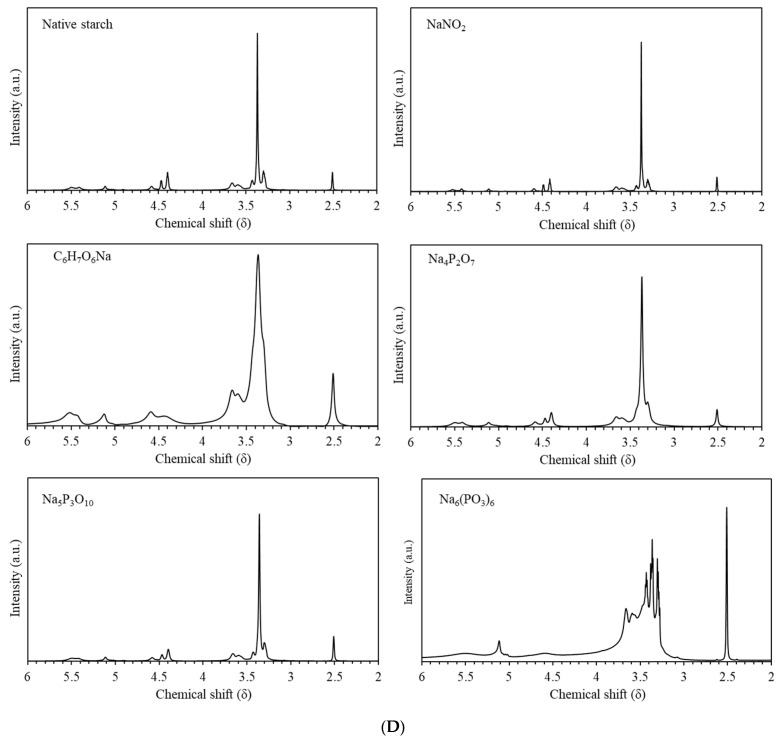
Chemical analysis, namely FTIR absorption spectra, between (**A**) 2500–3700 cm^−1^ and (**B**) 700–1400 cm^−1^, (**C**) intensity ratios of I_995_/I_1047_ and I_1047_/I_1035_ of IR peaks, and (**D**) ^1^H NMR spectra of native starch and starch extrudates containing food preservatives, namely tetrasodium pyrophosphate (Na_4_P_2_O_7_), sodium tripolyphosphate (Na_5_P_3_O_10_), sodium hexametaphosphate (Na_6_(PO_3_)_6_), sodium erythorbate (C_6_H_7_O_6_Na), and sodium nitrite (NaNO_2_). Different letters (a, b and c) indicate significant difference (*p* ≤ 0.05) between food preservatives.

Cassava starch is a sensitive structure susceptible to changes in its molecular order, including helix structure, chain size and conformation, and crystallinity [16] (Zhu, 2015). The α-(1,4) and α-(1,6) glycosidic linkages are essentially linked anhydroglucose units with high sensitivity to alterations in short-range molecular ordering, particularly near the α-(1,6) branches of amylopectin molecules [20,21] (Shi et al., 2007; Warren, Gidley & Flanagan, 2016). Figure 2A,B displays the FTIR absorption spectra of the TPS extrudates. The spectra exhibited two major absorption bands. A broad band at 3700–3000 cm^−1^ was attributed to stretching vibrations of free, inter-, and intra-molecular hydroxyl (O–H) groups. A band in the fingerprint region (1200–800 cm^−1^) was characteristic of starch extrudates [18,21]. The absorption bands at wavenumber 2914 cm^−1^ were ascribed to the asymmetric C–H stretching vibration. The highest IR absorption was found between 1300 and 950 cm^−1^ and represents asymmetric C–O stretching vibration. The absorption bands at 1147, 1080, and 974 cm^−1^ were assigned to stretching of C–O–C and C–O–H and C–O–H bending vibrations of glycosidic linkages of anhydroglucose units [18,19]. In addition, peaks at 889 and 1150–1300 cm^−1^ were assigned to asymmetric vibration stretching of P–O–C and symmetric P=O stretching vibration, respectively [18]. The analysis revealed modifications in the intensity of the broad hydroxyl group bands, particularly for starch containing 10% Na_5_P_3_O_10_. This suggests disruption of inter-, intra-, and free-molecular hydrogen bonds, possibly due to the substitution of hydroxyl groups in the anhydroglucose units by phosphate groups (PO_4_^3−^) from Na_5_P_3_O_10_, leading to a phosphorylation effect and increased P–O–C intensity. NaNO_2_ also displayed a lower hydrogen bonding intensity. This could be attributed to disruption of hydrogen bonds by nitric oxide (–N=O), leading to acid hydrolysis and nitrate ester (-ONO_2_) formation according to Dong and Vasanthan [18] and Shi, Zhang, Liu, Han, Zhang, Chen and Tian [20]. C_6_H_7_O_6_Na has a structure that overlaps with starch in the IR region, making it difficult to elucidate its specific interactions. However, a slight decrease in intensity at 995 cm^−1^ (assigned to intramolecular hydrogen bonding of C-6 hydroxyl groups in the starch unit) was observed, suggesting potential changes in starch structure [18,19,22].

Figure 2C presents the intensity ratios of specific IR peaks for the starch extrudates. The ratio between the intensities at 995 cm^−1^ and 1047 cm^−1^ (I_995_/I_1047_) reflects the degree of –OH groups at C-6 in the starch unit, using the C–O stretch of C–O–H in starch as a reference [7]. The results showed that increasing concentrations (2–10%) of Na_4_P_2_O_7_, Na_5_P_3_O_10_, Na_6_(PO_3_)_6_, C_6_H_7_O_6_Na, and NaNO_2_ slightly decreased the –OH bonding, indicating reduced levels of hydrogen bonding. Starch modification possibly occurred through phosphorylation (phosphate preservatives) or acid hydrolysis (C_6_H_7_O_6_Na and NaNO_2_). The intensity ratio between 1047 cm^−1^ and 1035 cm^−1^ reflects the ratio of short-ordered (crystalline) to amorphous structures in the starch, using the amorphous and crystalline regions from the anhydroglucose unit ring in cassava starch as a reference [18,19,20,22]. Increased concentrations of food preservatives (2–10%) led to a higher degree of amorphous structures in the starch extrudates. This suggests that the preservatives penetrated the starch chains, potentially through phosphorylation and acid hydrolysis, disrupting crystallinity during the melting process. These modifications to the starch chain conformation offer potential avenues for further development of functionally tailored starches.

### 3.3. Nuclear Magnetic Resonance (NMR)

Figure 2D shows the ^1^H NMR spectra of the native cassava starch and starch extrudates containing various food preservatives. The peak at 2.51 ppm corresponds to the solvent (DMSO-d6) used for the analysis [23]. The chemical shifts observed between 3.21 and 3.89 ppm represent the protons of the hydroxyl groups (HC–OH) at positions 2–6 in the anhydroglucose units [8,23,24]. Additionally, the peak at 4.46 ppm signifies the methylene protons (–CH_2_OCH_2_) of glycerol, the plasticizer used during extrusion. The analysis revealed that the incorporation of food preservatives did not alter the positions of the chemical shifts for the H2–H6 protons. However, a slight decrease and broadening of these peaks was observed, possibly due to the disruption of hydrogen bonds and starch chain interactions [16]. The signals between 4.51 and 5.61 ppm are attributed to the equatorial anomeric protons (H1) of the anhydroglucose units. The chemical shifts at around 5.10 ppm and 5.40–5.60 ppm correspond to H1 on the α-(1-6) linkages (branch points) and the α-(1-4) linkages within the anhydroglucose units, respectively [8,25]. Interestingly, these signals displayed a slight downfield shift (Figure S1) in the presence of specific food preservatives. Notably, C_6_H_7_O_6_Na and NaNO_2_ caused a shift in the α-(1-4) linkage peak, while Na_5_P_3_O_10_, Na_6_(PO_3_)_6_, C_6_H_7_O_6_Na, and NaNO_2_ all induced a shift in the α-(1-6) linkage peak. These findings suggest that phosphorylation and acid hydrolysis by Na_5_P_3_O_10_, Na_6_(PO_3_)_6_, C_6_H_7_O_6_Na, and NaNO_2_ might disrupt the α-(1-6) equatorial anomeric protons (H1). Additionally, C_6_H_7_O_6_Na and NaNO_2_ appear to hydrolyze the starch chains, leading to shorter chains through the disruption of α-(1-4) linkages.

### 3.4. Differential Scanning Calorimetry (DSC)

Table 1 summarizes the thermal behavior of the starch extrudates as determined by DSC analysis. The parameters include glass transition temperature (Tg), onset temperature (To), and peak temperature (Tp), which reflect the transitions within the semi-crystalline starch structure. The native extruded starch exhibited a glass transition temperature (Tg) of 67.08 °C, consistent with previous reports [7,18]. The incorporation of food preservatives into the extrudates influenced the mobility of starch and plasticizer chains, leading to variations in Tg. Na_4_P_2_O_7_ and NaNO_2_ decreased Tg, possibly due to the disruption of intermolecular hydrogen bonding between starch chains caused by hydrolysis reactions. Additionally, hydrolysis can generate shorter amylose segments and starch chains, increasing chain mobility and free volume within the amorphous region, potentially influencing recrystallization [7,18]. Concentrations of 2% Na_5_P_3_O_10_, Na_6_(PO_3_)_6_, and C_6_H_7_O_6_Na increased Tg (70–75 °C). This might be attributed to crosslinking and esterification reactions, leading to a more rigid and thermally stable structure [17,21]. To and Tp represent the onset and peak temperatures of the melting process for the crystalline regions of the starch (A, CA, and VH types, as confirmed by XRD). The To and Tp values for the starch-food preservative extrudates ranged from 185–193 °C, which were higher than those of the native starch (183.17 °C). Furthermore, these values tended to increase with higher concentrations of Na_5_P_3_O_10_ and Na_6_(PO_3_)_6_. This overall increase in To and Tp suggests that all food preservatives modified the starch conformation, potentially introducing covalent bonds between starch chains and ester or phosphate groups. These modifications could lead to longer starch chains and hinder recrystallization during the melting process. The incorporation of food preservatives effectively influenced the thermal properties of the starch extrudates. These effects were likely mediated by changes in chain conformation, mobility, and recrystallization behavior.

### 3.5. Thermogravimetric Analysis (TGA)

**Figure 3:**
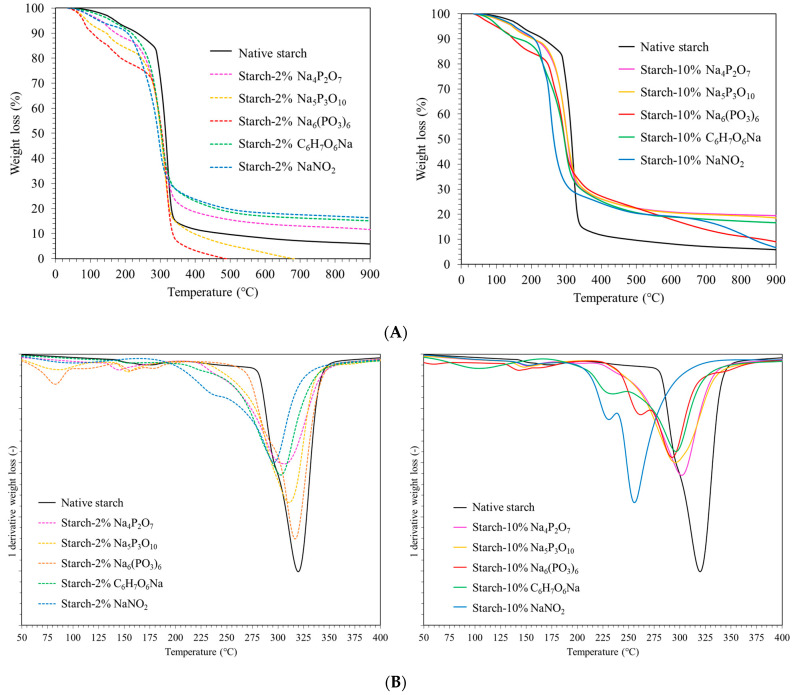
(**A**) Thermogravimetric curve and (**B**) 1st derivative TGA of native starch and starch extrudates containing food preservatives, namely tetrasodium pyrophosphate (Na_4_P_2_O_7_), sodium tripolyphosphate (Na_5_P_3_O_10_), sodium hexametaphosphate (Na_6_(PO_3_)_6_), sodium erythorbate (C_6_H_7_O_6_Na), and sodium nitrite (NaNO_2_).

Figure 3A depicts the TGA thermograms of the native starch and starch-food preservative extrudates. Two major weight-loss stages are observed. Stage 1 (100–200 °C): This stage corresponds to the dehydration of absorbed moisture and volatilization of glycerol (7–12% weight loss). Additionally, small-molecule-weight food preservatives, acting as plasticizers, may decompose in this range [26]. Stage 2 (290–350 °C): This major weight-loss stage (around 80%) represents the decomposition of amylose and amylopectin molecules [7,27]. The incorporation of food preservatives influenced the thermal stability of the extrudates, as reflected by slight changes in the residual mass percentage. After 350–900 °C, many samples still had about 20% of their weight remaining. This residual mass may be due to the complete absence or complete evaporation at 900 °C, or to the interaction between the food preservatives and the starch material, which can affect the thermal stability and decomposition behavior. Therefore, the residual mass after thermal processing is an advantage, indicating that the starch after extrusion is more stable. The rate of weight loss is related to the first derivative of the TGA curve, and the peak in this derivative curve indicates the degradation temperature (Td) of the polymer (Figure 3B). The temperature range between 50 and 200 °C shows that all food preservatives (Na_4_P_2_O_7_, Na_5_P_3_O_10_, Na_6_(PO_3_)_6_, C_6_H_7_O_6_Na, and NaNO_2_) shifted the decomposition temperature to lower values compared to the native starch extrudate. This suggests that food preservatives interact with the plasticizer-rich phase, potentially increasing chain mobility and reducing the interaction strength by disrupting hydrogen bonds. The Td of the native starch extrudate was 320 °C. Incorporation of 10% food preservative led to a more pronounced decrease in Td compared to 2% food preservative, particularly for starch-NaNO_2_. This reduction in Td is likely dependent on the concentration and type of reaction involved. For example, NaNO_2_ likely disrupts inter- and intra-molecular hydrogen bonds most effectively due to hydrolysis, while Na_4_P_2_O_7_, Na_5_P_3_O_10_, and Na_6_(PO_3_)_6_ might introduce phosphate groups through phosphorylation, also affecting hydrogen bonding. The disruption of hydrogen bonding by food preservatives appears to be a key factor influencing the thermal stability and decomposition of the starch extrudates.

### 3.6. Water Absorption Capacity

**Figure 4:**
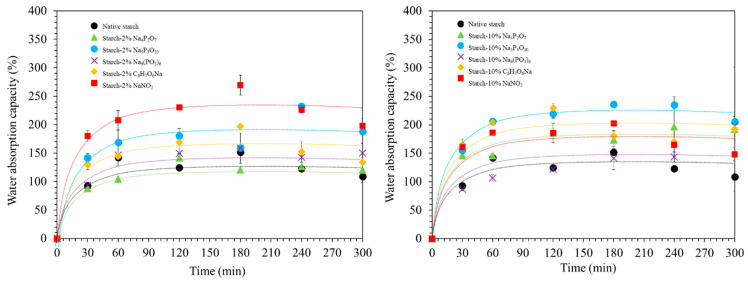
Water absorption capacity of native starch and starch extrudates containing food preservatives, namely tetrasodium pyrophosphate (Na_4_P_2_O_7_), sodium tripolyphosphate (Na_5_P_3_O_10_), sodium hexametaphosphate (Na_6_(PO_3_)_6_), sodium erythorbate (C_6_H_7_O_6_Na), and sodium nitrite (NaNO_2_).

Water absorption is a crucial property of starch-based materials, particularly for applications in food, pharmaceuticals, and biomaterials. Understanding this property can provide valuable insights into the material’s behavior and potential applications. Figure 4 illustrates the water absorption behavior of the native cassava starch and starch-food preservative extrudates. Water absorption capacity reflects the ability of a material to retain water, which is determined by the interactions between starch and water molecules. Several factors influence water absorption, including the composition of the starch, its granular microstructure, long-range crystalline structures, short-range ordered structures, and the presence of hydrophilic groups [28,29]. The results showed that the incorporation of food preservatives generally increased the water absorption capacity of the starch extrudates compared to the native starch. Initially, water absorption increased rapidly, reaching an equilibrium around 60 min. However, prolonged exposure (up to 300 min) led to a decrease in water absorption capacity, possibly due to increased starch dissolution [30]. At 60 min (as shown in Figure S2), starch extrudates containing 2% NaNO_2_, 2% Na_5_P_3_O_10_, 2% C_6_H_7_O_6_Na, and 2% Na_6_(PO_3_)_6_ exhibited increased water absorption capacity. This can be attributed to the presence of hydrophilic groups (P=O, −OH, and N=O) in these food preservatives, which enhance the overall hydrophilicity of the starch and facilitate water penetration into the structure. Conversely, a 2% Na_4_P_2_O_7_ addition resulted in a decrease in water absorption. This might be due to the increased degree of crystallinity in the starch, hindering water penetration. Interestingly, the water absorption capacity of starch extrudates containing 10% NaNO_2_ and 10% Na_6_(PO_3_)_6_ showed a slight decrease. This could be related to the higher proportion of short-range ordered structures observed in the FTIR analysis (Figure 2C, I_1047_/I_1035_ ratio). These more ordered starch granules might restrict water penetration, causing it to move inwards rather than being readily absorbed. The water absorption behavior of the starch extrudates was significantly influenced by the chemical nature of the food preservatives and the crystallinity of the starch. Hydrophilic groups in food preservatives generally enhanced water absorption, while increased crystallinity could hinder it.

### 3.7. Water Solubility

**Figure 5:**
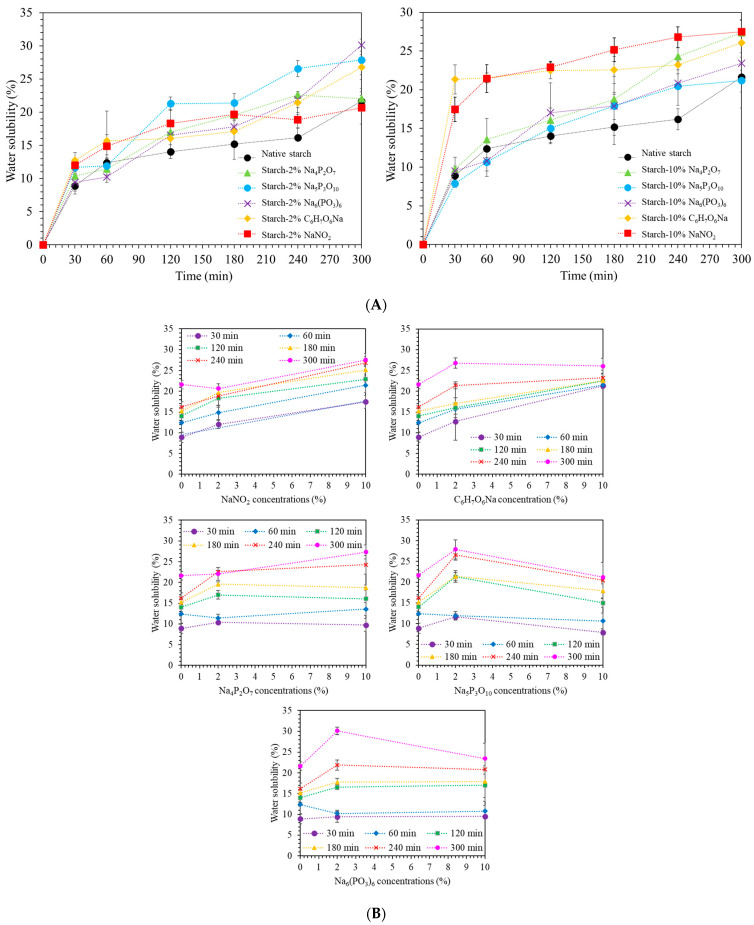

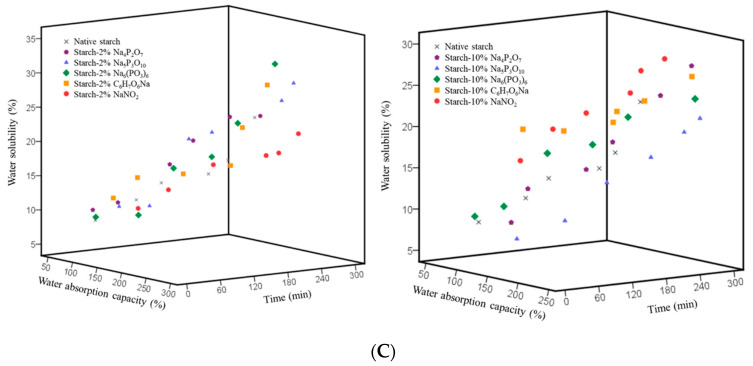
(**A**) Water solubility, (**B**) water solubility as a function of food preservative concentration at different time intervals (30, 60, 120, 180, 240 and 300 min) and (**C**) the relationship between water absorption, water solubility, and time for native starch and starch extrudates containing food preservatives, namely tetrasodium pyrophosphate (Na_4_P_2_O_7_), sodium tripolyphosphate (Na_5_P_3_O_10_), sodium hexametaphosphate (Na_6_(PO_3_)_6_), sodium erythorbate (C_6_H_7_O_6_Na), and sodium nitrite (NaNO_2_).

Water solubility is a crucial parameter influencing the functional properties of starch extrudates. Figure 5A depicts the water solubility of native starch and starch-food preservative extrudates as a function of time. As expected, water solubility increased for all samples over time. The water solubility followed this trend: NaNO_2_ and C_6_H_7_O_6_Na > Na_4_P_2_O_7_, Na_5_P_3_O_10_, and Na_6_(PO_3_)_6_. The enhanced solubility observed with NaNO_2_ and C_6_H_7_O_6_Na can be attributed to several factors. First, small molecular weight: due to their smaller size compared to starch molecules, NaNO_2_ and C_6_H_7_O_6_Na can more easily penetrate the intricate structure of the starch, facilitating interaction with water molecules within the matrix. Second, the presence of hydrophilic groups (like -OH in hydroxyl groups and N=O in nitrite) in these preservatives allows them to form hydrogen bonds with water molecules. This creates a bridge between the water and the starch, promoting water absorption and ultimately increasing solubility. Third, NaNO_2_ and C_6_H_7_O_6_Na can disrupt the existing hydrogen bonds between starch molecules. These hydrogen bonds play a crucial role in maintaining the compact structure of starch granules. By disrupting them, the starch structure becomes looser and more accessible to water molecules, leading to increased water solubility [7,31,32]. Figure 5B explores the relationship between water solubility and food preservative concentration at different time points. NaNO_2_ and C_6_H_7_O_6_Na consistently increased water solubility at all concentrations, likely due to their ability to disrupt hydrogen bonding in the starch. While the presence of phosphate groups (P=O) in Na_4_P_2_O_7_, Na_5_P_3_O_10_, and Na_6_(PO_3_)_6_ generally enhances water solubility, increasing their concentration led to a decrease in water solubility [1]. This suggests the formation of crosslinks between starch molecules by phosphates, hindering water penetration and dissolution [33]. Notably, Na_5_P_3_O_10_ appeared to be the most effective in crosslinking, resulting in the lowest water solubility.

Furthermore, Figure 5C demonstrates the correlation between water absorption capacity, water solubility, and time. It reveals that higher water absorption capacity coincides with increased water solubility. Interestingly, the concentration of phosphate-based preservatives had the least significant impact on solubility. This suggests that these preservatives, even at higher concentrations (10%), might not be present in sufficient quantities to form extensive crosslinks throughout the starch structure. The water solubility of the starch extrudates was significantly influenced by the type and concentration of the incorporated food preservative. NaNO_2_ and C_6_H_7_O_6_Na exhibited superior solubility-enhancing effects due to their ability to disrupt hydrogen bonding. In addition, starch-NaNO_2_ systems with the lowest crystallinity exhibit the highest water absorption, and solution capacity can be attributed to crystallinity-amorphous regions, interaction between compound and matrix, hydrogen bonding, and polymer hydrophilicity. Lower crystallinity implies a higher proportion of amorphous regions within the starch structure. These amorphous regions are generally more accessible to water molecules due to their less ordered arrangement, leading to higher water absorption and solution capacity. NaNO_2_ can interact with both the crystalline and amorphous regions of starch. However, its interaction with the amorphous regions may be more pronounced, leading to increased disruption of the starch structure and enhanced water uptake [7,30,31,32]. Conversely, phosphate-based preservatives, especially at higher concentrations, might form crosslinks within the starch matrix, leading to decreased water solubility. The results suggested that the addition of food preservatives has an effect on water absorption and water solubility, which can be applied in the pharmaceutical, food, and non-food industries due to their different solubility.

### 3.8. Antimicrobial Analysis of TPS Extrudates Blended PBAT as Active Films

**Figure 6:**
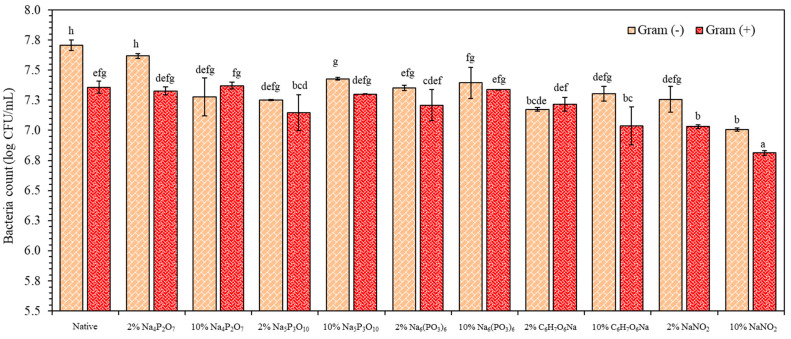
Antimicrobial analysis as total plate count after 24 h of incubation of the native and starch extrudates containing food preservatives, namely tetrasodium pyrophosphate (Na_4_P_2_O_7_), sodium tripolyphosphate (Na_5_P_3_O_10_), sodium hexametaphosphate (Na_6_(PO_3_)_6_), sodium erythorbate (C_6_H_7_O_6_Na), and sodium nitrite (NaNO_2_), blended PBAT films against *Escherichia coli* (gram (−)) and *Staphylococcus aureus* (gram (+)). Different letters (a-h) indicate significant difference (*p* ≤ 0.05) between food preservatives.

The native starch and starch containing 2% and 10% of each food preservative were blended with PBAT polyester to produce active films via single-screw blown film extrusion (Figure S3). The active films were analyzed for antimicrobial activity by total plate count against *Escherichia coli* and *Staphylococcus aureus*, Gram-negative and Gram-positive bacteria, respectively (Figure 6). Incorporation of each food preservative exhibited a reduction in the number of both Gram-positive and Gram-negative bacteria. Increasing the concentration from 2% to 10% of Na_5_P_3_O_10_ and Na_6_(PO_3_)_6_ did not significantly increase the colony count of either bacteria. Na_4_P_2_O_7_, C_6_H_7_O_6_Na, and NaNO_2_ demonstrated effective antimicrobial activity, likely due to the higher antimicrobial efficiency of C_6_H_7_O_6_Na and NaNO_2_ compared to phosphate groups. NaNO_2_ primarily produces nitric oxide (NO) through enzymatic and non-enzymatic pathways, which destroys the cell membranes of bacteria [34,35,36]. Phosphate groups interacted with bacterial cells by binding with metal ions, leading to changes in cell wall structure and inhibiting microbial growth. This resulted in a lower effective antimicrobial efficiency, with insignificant antimicrobial activity. However, the antimicrobial capacity for Gram-negative bacteria was generally higher than for Gram-positive bacteria, possibly because Gram-negative bacteria have thinner cell walls, making them more susceptible to chemicals [34,37]. Therefore, the development of starch extrudates into active films can enhance antibacterial performance, providing an alternative function for starch extrudates in both food and packaging applications such as meat, shrimp, and fresh-cut active films [7,14,38,39,40].

## 4. Conclusions

This study investigated the effects of incorporating various food preservatives (Na_4_P_2_O_7_, Na_5_P_3_O_10_, Na_6_(PO_3_)_6_, C_6_H_7_O_6_Na, and NaNO_2_) into cassava starch using thermal twin-screw extrusion. ^1^H NMR analysis revealed the disruption of hydrogen bonding within the starch molecule, particularly affecting hydroxyl groups, by all food preservatives. Na_4_P_2_O_7_, Na_5_P_3_O_10_, and Na_6_(PO_3_)_6_ modified starch through phosphorylation reactions, while C_6_H_7_O_6_Na and NaNO_2_ additionally induced starch chain hydrolysis, leading to shorter chain lengths. This was confirmed by ATR-FTIR and ^1^H NMR analyses. These modifications in hydrogen bonding and chain length likely influenced the degree of crystallization in the starch extrudates. The disruption of hydrogen bonds within the anhydroglucose units increased the amorphous regions within the starch structure. The interaction between food preservatives and the plasticizer-rich phase, as well as the food preservative-starch-rich phase, reduced hydrogen bond strength and improved chain mobility. This resulted in a decrease in thermal transition temperatures and Td as observed in DSC and TGA analyses. Food preservatives generally increased the water absorption capacity of the starch extrudates. Notably, C_6_H_7_O_6_Na and NaNO_2_ exhibited the most significant enhancement in water solubility. This could be attributed to their ability to disrupt hydrogen bonding within the starch, facilitating water interaction. The incorporation of food preservatives during thermal extrusion effectively modified the cassava starch extrudates. These modifications included changes in conformation, physical properties, and thermal behavior. The increase in water absorption and solubility, particularly with C_6_H_7_O_6_Na and NaNO_2_, suggests potential applications in food formulations where these properties are desirable.

## Figures and Tables

**Table 1:** Glass transition temperature (Tg), onset temperature (To), and peak temperature (Tp) as determined by differential scanning calorimetry (DSC) of native starch and starch extrudates containing food preservatives, namely tetrasodium pyrophosphate (Na_4_P_2_O_7_), sodium tripolyphosphate (Na_5_P_3_O_10_), sodium hexametaphosphate (Na_6_(PO_3_)_6_), sodium erythorbate (C_6_H_7_O_6_Na), and sodium nitrite (NaNO_2_).

Samples	T_g_ (°C)	T_o_ (°C)	T_p_ (°C)
Native starch	67.08	180.60	183.17
Starch-2% Na_4_P_2_O_7_	56.17	182.66	187.50
Starch-10% Na_4_P_2_O_7_	66.75	182.35	185.33
Starch-2% Na_5_P_3_O_10_	74.43	179.24	183.00
Starch-10% Na_5_P_3_O_10_	71.59	190.26	193.17
Starch-2% Na_6_(PO_3_)_6_	71.98	179.13	182.17
Starch-10% Na_6_(PO_3_)_6_	66.87	183.83	186.00
Starch-2% C_6_H_7_O_6_Na	70.98	182.77	187.17
Starch-10% C_6_H_7_O_6_Na	67.46	174.77	178.50
Starch-2% NaNO_2_	66.07	183.52	187.50
Starch-10% NaNO_2_	49.88	183.87	186.50

## Data Availability

The original contributions presented in the study are included in the article/Supplementary Materials, further inquiries can be directed to the corresponding author.

## Supplementary Materials

The following supporting information can be downloaded at https://www.mdpi.com/article/10.3390/polym16192787/s1: Figure S1: ^1^H NMR spectra (δ = 5–6 ppm) showing glycosidic linkage disruption; Figure S2: Water absorption capacity; Figure S3: The native starch and starch containing 2% and 10% of each food preservative were blended with PBAT to produce active films via single-screw blown film extrusion.

## References

[1] Leonel M., Del Bem M.S., Dos Santos T.P., Franco C.M.L. (2021). Preparation and properties of phosphate starches from tuberous roots. Int. J. Biol. Macromol..

[2] Xu M., Xu C., Kim S.-J., Ji S., Ren Y., Chen Z., Li Y., Zhou B., Lu B. (2024). Investigating the evolution of the fine structure in cassava starch during growth and its correlation with gelatinization performance. Int. J. Biol. Macromol..

[3] Ferreira L.F., de Oliveira A.C.S., de Oliveira Begali D., de Sena Neto A.R., Martins M.A., de Oliveira J.E., Borges S.V., Yoshida M.I., Tonoli G.H.D., Dias M.V. (2021). Characterization of cassava starch/soy protein isolate blends obtained by extrusion and thermocompression. Ind. Crops Prod..

[4] Wang L., Xu J., Zhang M., Zheng H., Li L. (2022). Preservation of soy protein-based meat analogues by using PLA/PBAT antimicrobial packaging film. Food Chem..

[5] Lin R.H., Fan Y.Y., Liu T., Yang H., Ma L.J., Huang X.J., Liu Y. (2020). Structural characterization of controlled decrystallization of cassava starch. Starch-Stärke.

[6] Qi M., Song J., Jiang L., Li L., Xu M., Li Y., Yu S., Li H. (2024). Understanding the degradation mechanisms of cyanide and starch in cassava flour during extrusion processing. Innov. Food Sci. Emerg. Technol..

[7] Wongphan P., Nerin C., Harnkarnsujarit N. (2023). Enhanced compatibility and functionality of thermoplastic cassava starch blended PBAT blown films with erythorbate and nitrite. Food Chem..

[8] Chang C.C., Trinh B.M., Mekonnen T.H. (2021). Robust multiphase and multilayer starch/polymer (TPS/PBAT) film with simultaneous oxygen/moisture barrier properties. J. Colloid Interface Sci..

[9] San H., Harnkarnsujarit N. (2023). Sulfite incorporated thermoplastic cassava starch blended PBAT blown films as antimicrobial and antibrowning packaging. Ind. Crops Prod..

[10] Varghese S.A., Phothisarattana D., Srisa A., Laorenza Y., Jarupan L., Bumbudsanpharoke N., Chonhenchob V., Harnkarnsujarit N. (2023). Novel eco-friendly antimicrobial UV-blocking PBAT/PBS/TiO_2_ nanocomposite films for improved shelf-life of bananas. Food Biosci..

[11] Nisitthichai J., Wannaphruek P., Sriprablom J., Suphantharika M., Smith S.M., Amornsakchai T., Wongsagonsup R. (2024). Impact of Oil Addition on Physicochemical Properties and In Vitro Digestibility of Extruded Pineapple Stem Starch. Polymers.

[12] Zhang Y., Shao F., Wan X., Zhang H., Cai M., Hu K., Duan Y. (2024). Effects of rapeseed protein addition on soybean protein-based textured protein produced by low-moisture extrusion: Changes in physicochemical attributes, structural properties and barrel flow behaviors. Food Hydrocoll..

[13] Wongphan P., Panrong T., Harnkarnsujarit N. (2022). Effect of different modified starches on physical, morphological, thermomechanical, barrier and biodegradation properties of cassava starch and polybutylene adipate terephthalate blend film. Food Packag. Shelf Life.

[14] Laorenza Y., Harnkarnsujarit N. (2021). Carvacrol, citral and α-terpineol essential oil incorporated biodegradable films for functional active packaging of Pacific white shrimp. Food Chem..

[15] Mutungi C., Passauer L., Onyango C., Jaros D., Rohm H. (2012). Debranched cassava starch crystallinity determination by Raman spectroscopy: Correlation of features in Raman spectra with X-ray diffraction and ^13^C CP/MAS NMR spectroscopy. Carbohydr. Polym..

[16] Zhu F. (2015). Composition, structure, physicochemical properties, and modifications of cassava starch. Carbohydr. Polym..

[17] Liu Y., Fan L., Mo X., Yang F., Pang J. (2018). Effects of nanosilica on retrogradation properties and structures of thermoplastic cassava starch. J. Appl. Polym. Sci..

[18] Dong H., Vasanthan T. (2020). Effect of phosphorylation techniques on structural, thermal, and pasting properties of pulse starches in comparison with corn starch. Food Hydrocoll..

[19] Pozo C., Rodríguez-Llamazares S., Bouza R., Barral L., Castaño J., Müller N., Restrepo I. (2018). Study of the structural order of native starch granules using combined FTIR and XRD analysis. J. Polym. Res..

[20] Shi R., Zhang Z., Liu Q., Han Y., Zhang L., Chen D., Tian W. (2007). Characterization of citric acid/glycerol co-plasticized thermoplastic starch prepared by melt blending. Carbohydr. Polym..

[21] Warren F.J., Gidley M.J., Flanagan B.M. (2016). Infrared spectroscopy as a tool to characterise starch ordered structure--a joint FTIR-ATR, NMR, XRD and DSC study. Carbohydr. Polym..

[22] Si W., Weng Y., Tan B., Zhang S. (2022). Adopted ion-pair effect to construct bicontinuous starch-based gel and its application in humidity sensitivity and strain-responsiveness. Compos. Part B Eng..

[23] Tizzotti M.J., Sweedman M.C., Tang D., Schaefer C., Gilbert R.G. (2011). New 1H NMR procedure for the characterization of native and modified food-grade starches. J. Agric. Food Chem..

[24] De Graaf R., Lammers G., Janssen L., Beenackers A. (1995). Quantitative analysis of chemically modified starches by ^1^H-NMR spectroscopy. Starch-Stärke.

[25] Xu A., Seib P. (1997). Determination of the Level and Position of Substitution in Hydroxypropylated Starch by High-Resolution1H-NMR Spectroscopy ofAlpha-limit Dextrins. J. Cereal Sci..

[26] Lendvai L., Apostolov A., Karger-Kocsis J. (2017). Characterization of layered silicate-reinforced blends of thermoplastic starch (TPS) and poly(butylene adipate-co-terephthalate). Carbohydr. Polym..

[27] Teodoro A.P., Mali S., Romero N., de Carvalho G.M. (2015). Cassava starch films containing acetylated starch nanoparticles as reinforcement: Physical and mechanical characterization. Carbohydr. Polym..

[28] Wang Y.R., Zhang B., Fan J.L., Yang Q., Chen H.Q. (2019). Effects of sodium tripolyphosphate modification on the structural, functional, and rheological properties of rice glutelin. Food Chem..

[29] Zhang J., Tao L., Yang S., Li Y., Wu Q., Song S., Yu L. (2023). Water absorption behavior of starch: A review of its determination methods, influencing factors, directional modification, and food applications. Trends Food Sci. Technol..

[30] Zeng J.-B., Jiao L., Li Y.-D., Srinivasan M., Li T., Wang Y.-Z. (2011). Bio-based blends of starch and poly(butylene succinate) with improved miscibility, mechanical properties, and reduced water absorption. Carbohydr. Polym..

[31] Jaekel L.Z., Schmiele M., da Silva Rodrigues R., Chang Y.K. (2015). Influence of the extrusion process on the technological properties of hydroxypropylated cross-linked cassava starch. J. Food Sci. Technol..

[32] Sangokunle O.O., Sathe S.K., Singh P. (2020). Purified starches from 18 pulses have markedly different morphology, oil absorption and water absorption capacities, swelling power, and turbidity. Starch-Stärke.

[33] Ashogbon A.O., Akintayo E.T. (2014). Recent trend in the physical and chemical modification of starches from different botanical sources: A review. Starch-Stärke.

[34] Nateghi L., Zarei F., Afshari K.P. (2024). The Effect of Sodium Nitrite Replacement with Lycopene Pigment in German Sausage and Evaluation of Its Physicochemical, Antimicrobial and Sensory Properties. J. Nutr. Food Secur..

[35] Chatkitanan T., Harnkarnsujarit N. (2021). Effects of nitrite incorporated active films on quality of pork. Meat Sci..

[36] Karpińska-Tymoszczyk M. (2013). The effect of oil-soluble rosemary extract, sodium erythorbate, their mixture, and packaging method on the quality of Turkey meatballs. J. Food Sci. Technol..

[37] Laorenza Y., Harnkarnsujarit N. (2023). Ginger oil and lime peel oil loaded PBAT/PLA via cast-extrusion as shrimp active packaging: Microbial and melanosis inhibition. Food Packag. Shelf Life.

[38] Leelaphiwat P., Pechprankan C., Siripho P., Bumbudsanpharoke N., Harnkarnsujarit N. (2022). Effects of nisin and EDTA on morphology and properties of thermoplastic starch and PBAT biodegradable films for meat packaging. Food Chem..

[39] Phothisarattana D., Wongphan P., Promhuad K., Promsorn J., Harnkarnsujarit N. (2021). Biodegradable Poly(Butylene Adipate-Co-Terephthalate) and Thermoplastic Starch-Blended TiO2 Nanocomposite Blown Films as Functional Active Packaging of Fresh Fruit. Polymers.

[40] Phothisarattana D., Wongphan P., Promhuad K., Promsorn J., Harnkarnsujarit N. (2022). Blown film extrusion of PBAT/TPS/ZnO nanocomposites for shelf-life extension of meat packaging. Colloids Surf. B Biointerfaces.

