# Supplementary material for: Modifying Cassava Starch via Extrusion with Phosphate, Erythorbate and Nitrite: Phosphorylation, Hydrolysis and Plasticization
Source: Polymers (Basel). 2024 Oct 1;16(19):2787. doi: 10.3390/polym16192787 (PMC11478379; doi:10.3390/polym16192787)
Supplement: Supplementary file 1 [file polymers-16-02787-s001.zip › polymers-3203708-supplementary.pdf]

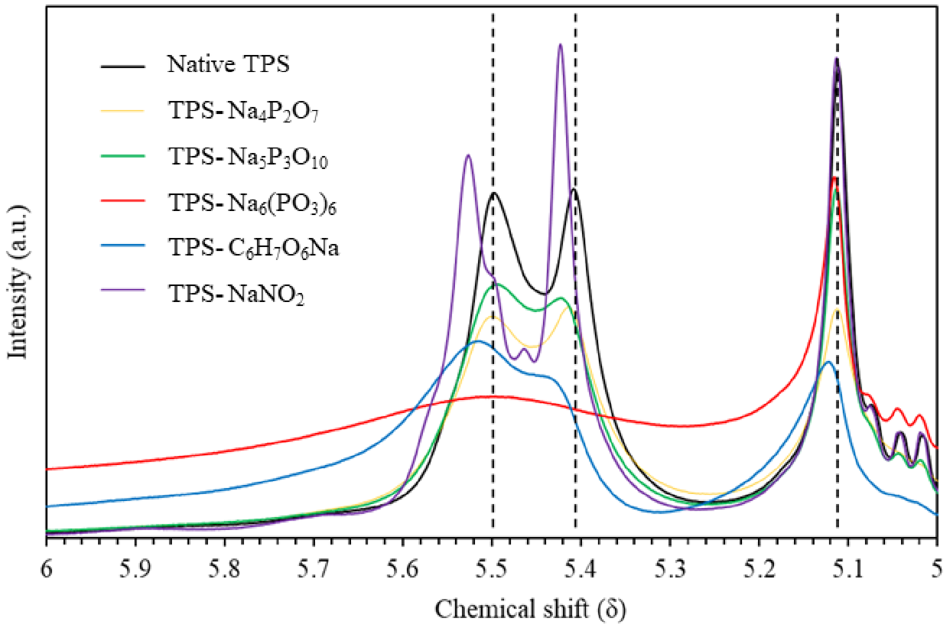

Figure S1 ^{1}H NMR spectra (δ=5-6 ppm) showing glycosidic linkage disruption of native starch and starch extrudates containing food preservatives namely tetrasodium pyrophosphate ($Na_4P_2O_7$), sodium tripolyphosphate ($Na_5P_3O_{10}$), sodium hexametaphosphate ($Na_6(PO_3)_6$), sodium erythorbate ($C_6H_7O_6Na$) and sodium nitrite ($NaNO_2$).

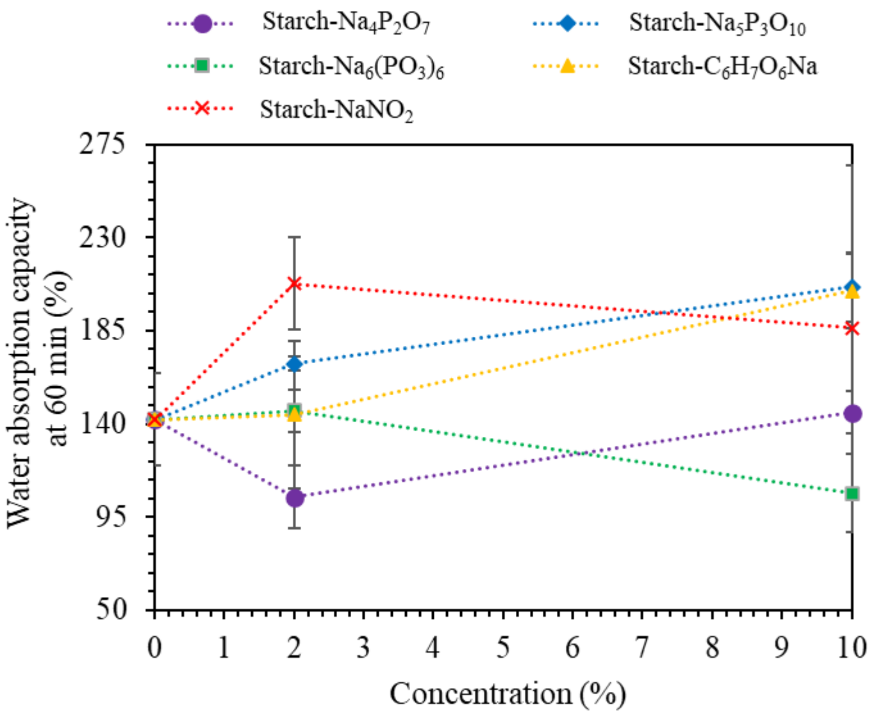

Figure S2 Water absorption capacity of native starch and starch extrudates containing food preservatives namely tetrasodium pyrophosphate ($Na_4P_2O_7$), sodium tripolyphosphate ($Na_5P_3O_{10}$), sodium hexametaphosphate ($Na_6(PO_3)_6$), sodium erythorbate ($C_6H_7O_6Na$) and sodium nitrite ($NaNO_2$).

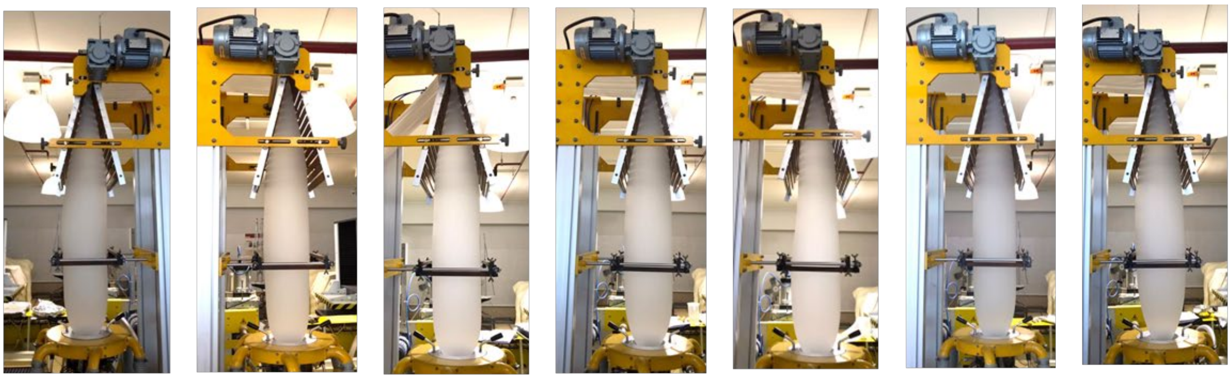

Native starch/PBAT | Starch-2% $Na_4P_2O_7$/PBAT | Starch-10% $Na_4P_2O_7$/PBAT | Starch-2%$Na_5P_3O_{10}$/PBAT | Starch-10%$Na_5P_3O_{10}$/PBAT | Starch-2% $Na_6(PO_3)_6$/PBAT | Starch-10% $Na_6(PO_3)_6$/PBAT

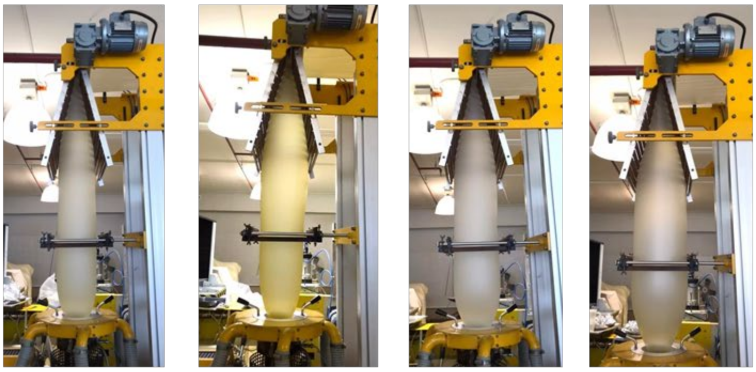

Starch-2% $C_6H_7O_6Na$ PBAT | Starch-10% $C_6H_7O_6Na$ PBAT | Starch-2% $NaNO_2$/PBAT | Starch-10% $NaNO_2$/PBAT

Figure S3 The native starch and starch containing 2% and 10% of each food preservative were blended with PBAT to produce active films via single screw blown film extrusion.
